# Greater improvement in counting Stroop accuracy following a positive interpersonal and life orientation training program: a non-randomized controlled study in Taiwanese adolescents

**DOI:** 10.3389/fpsyg.2026.1966721

**Published:** 2026-10-05

**Authors:** Chia-Yu Cheng, April Chiung-Tao Shen, Wen-Sung Lai, Chiao-Yu Yang, Deng-Min Chuang, Yu-An Lin, Tai-Li Chou

**Affiliations:** 1 Department of Psychology, National Taiwan University, Taipei, Taiwan; 2 NTU Children and Family Research Center Sponsored by CTBC Charity Foundation, Taipei, Taiwan; 3 Department of Social Work, National Taiwan University, Taipei, Taiwan; 4 School of Social Work, Morgan State University, Baltimore, MD, United States; 5 Graduate Institute of Social Work, National Taiwan Normal University, Taipei, Taiwan; 6 Department of Social Work, National Taipei University, New Taipei, Taiwan

**Keywords:** counting Stroop accuracy, counting Stroop task, positive interpersonal and life orientation training (PILOT), psychological well-being, psychological well-being scale

## Abstract

Adolescence presents a unique developmental challenge, specifically around inhibitory control (IC), a crucial executive function that, when underdeveloped, strongly predicts negative trajectories like poor academic performance and psychopathology. Our intervention-based study, utilizing the culturally relevant, school-based life skill training, the Positive Interpersonal and Life Orientation Training (PILOT) program, targeted 678 7th-grade students over 12 months to assess its impact on IC and psychological well-being in the PILOT group (*n* = 404) and the control group (*n* = 274). Our findings were crucial: the PILOT group showed greater pre-to-post improvement in overall counting Stroop accuracy than the control group. Of the six domains of psychological well-being assessed, the two groups differed on one: self-acceptance declined in the control group but did not change in the PILOT group. Within the PILOT group, change in Stroop interference was associated with concurrent change in three well-being domains. More critically, these observed gains in IC were a significant predictor of subsequent improvements across four critical domains of well-being: Autonomy, Environmental Mastery, Purpose in Life, and Self-Acceptance. These data indicate that a school-based life skills program delivered in ordinary classrooms was accompanied by greater improvement in counting Stroop accuracy, and that its relation to self-reported psychological well-being was confined to the maintenance of self-acceptance. These data indicate that targeted life skills training may serve as a fundamental capacity underpinning broader emotional and personal growth during this critical developmental period

## 1 Introduction

Adolescence is a critical developmental stage marked by profound emotional, cognitive, and social changes, alongside the rapid formation and refinement of neural connections in the brain (Silvers, 2022). This period of heightened neuroplasticity supports adolescents’ capacity to learn and adjust to evolving social roles and environments. However, the dynamic interaction between ongoing brain development and external social influences can contribute to difficulties in cognitive regulation and behavioral control (Andrews et al., 2021). If left unaddressed, these challenges may negatively impact various domains, including academic achievement (Ivcevic and Brackett, 2014), social functioning (Chervonsky and Hunt, 2019), susceptibility to substance use (Wills et al., 2011), and overall psychological well-being (Yousefi Afrashteh and Hasani, 2022). These developmental disruptions not only affect adolescents’ immediate well-being but may also pose long-term risks to mental health and social adjustment. Therefore, supporting adolescents in strengthening their capacity to manage thoughts, emotions, and behaviors is essential to fostering resilience and minimizing the likelihood of maladaptive outcomes (Moltrecht et al., 2021).

### 1.1 The importance of inhibitory control in adolescence

Inhibitory control is particularly important during adolescence, as it supports the ability to suppress impulsive responses and resist distractions—functions that contribute to the broader regulation of attention, emotion, and behavior. This ability becomes increasingly essential as adolescents encounter complex cognitive and social demands, including greater susceptibility to peer pressure and expanding autonomy in decision-making—factors that further challenge their developing self-regulatory systems (Steinberg, 2010; Crone and Dahl, 2012). When underdeveloped, inhibitory control is associated with a range of adverse outcomes, including academic underachievement, externalizing behaviors such as aggression and delinquency, and increased vulnerability to psychopathologies, including anxiety and depression (Moffitt et al., 2011; Wills et al., 2011). Longitudinal research has shown that lower levels of inhibitory control in early adolescence predict greater increases in delinquent behavior over time (Fosco et al., 2019). Conversely, adolescents with stronger inhibitory control are generally more capable of resisting impulsive urges, managing emotional responses, and maintaining focus on long-term objectives (Diamond, 2013).

Inhibitory control not only contributes to academic achievement and healthy social functioning but is also strongly linked to psychological well-being. Research indicates that adolescents and young adults with higher non-emotional inhibitory control tend to exhibit greater psychological resilience, while those with better emotional inhibitory control report fewer symptoms of anxiety and distress in challenging contexts (Afek et al., 2021). Similarly, a study on older adults found that those with more efficient inhibitory control, as reflected by quicker interference times, experienced higher levels of perceived personal growth—an essential aspect of psychological well-being (Fusi et al., 2022). These findings collectively highlight that inhibitory control supports both short-term emotional regulation and long-term psychological development. Strengthening this capacity may therefore serve as a buffer against mental health difficulties, enhancing resilience and emotional stability throughout life.

Intervention studies aimed at improving inhibitory control often use response inhibition training, particularly the Go/No-Go (GNG) and Stop-Signal Task (SST) paradigms. Evidence shows that GNG training alone, or in combination with SST, leads to immediate improvements in cognitive function among healthy adults. In contrast, SST training alone does not produce significant short-term gains. However, research also indicates that these improvements are not sustained over time when using either approach in isolation. Notably, the combination of GNG and SST training appears more promising for achieving longer-lasting effects, likely due to their complementary mechanisms: GNG taps into fast, automatic inhibition, while SST engages slower, top-down control (Li et al., 2022). Despite this, improvements from combined training may still fall short of producing meaningful changes in real-life behavior unless situated within more comprehensive, real-world interventions. Zelazo et al. (2024) emphasize that executive function training is more likely to generalize (i.e., transfer effectively to new and complex situations) when embedded within broader life skills programs. Such frameworks offer adolescents regular opportunities to practice cognitive and emotional regulation strategies in everyday settings, thereby promoting more sustained and functional self-regulation. Recognizing the limitations of purely cognitive approaches, many researchers now advocate for integrative models such as Life Skills Training (LST) to enhance adolescents’ self-regulatory capacities.

### 1.2 Life skills training and positive interpersonal and life orientation training

According to the World Health Organization (1999), life skills are defined as “abilities for adaptive and positive behavior that enable individuals to effectively navigate the demands and challenges of everyday life.” Life skills training programs are designed to strengthen these competencies by providing individuals with a core set of essential abilities (Gerami et al., 2015).

Systematic reviews have demonstrated a range of positive outcomes from these programs, particularly among adolescents. Benefits include reductions in substance use (Nasheeda et al., 2019; Yang et al., 2025), improved psychological well-being (Wu et al., 2023), increased self-esteem, and better emotional regulation, along with fewer symptoms of depression, anxiety, and stress (Swamy et al., 2024). For example, Lee et al. (2020) adapted the Life Skills Training (LST) program to include locally relevant scenarios from Taiwanese children’s daily experiences. Their findings revealed improvements in participants’ emotional regulation and reductions in depressive symptoms. Moreover, Naqvi and Chandra (2025) emphasized the bidirectional relationship between cognitive functioning and life skills: cognitive abilities facilitate the acquisition of adaptive behaviors, while participation in life skills training further enhances cognitive development. This highlights the importance of a holistic framework that integrates both cognitive and behavioral growth in adolescent development.

In this study, we adapted the conventional life skills framework into a culturally responsive program known as PILOT (Positive Interpersonal and Life Orientation Training). PILOT was specifically developed to align with the Taiwanese educational system, enhancing both its practicality and cultural relevance for implementation within school settings. The program outlines its core competencies, thematic modules, and learning objectives in detailed teacher and student manuals, enabling seamless integration into regular classroom instruction. Compared to traditional Life Skills Training (LST), PILOT introduces three key innovations that set it apart (Wu et al., 2023):

1) Broader Emphasis on Positive Child/Youth Development: Unlike traditional programs that primarily aim to prevent negative outcomes such as emotional distress or risky behaviors, PILOT adopts a strength-based approach rooted in positive psychology. It incorporates evidence-based practices like mindful meditation to actively cultivate resilience, psychological flourishing, and self-efficacy in resisting substances. This shift reflects a proactive model of adolescent development, emphasizing well-being enhancement rather than merely problem reduction.
2) Improved Curriculum and Teacher Support: Unlike standard LST programs, PILOT was refined using teacher feedback to ensure the curriculum is more engaging and practical. It features a comprehensive course structure and provides teachers with hands-on workshops, enabling them to effectively integrate positive psychology strategies into everyday classroom activities.
3) Contextual Validation and Focus on Implementation Quality: PILOT places a strong emphasis on implementation fidelity, and a previous evaluation assessed intervention exposure from students’ reports of how many sessions they had received (Wu et al., 2023). Its application across different phases—both before and after the COVID-19 pandemic—has provided important insights into how variations in delivery quality, whether in online or face-to-face formats, influence program effectiveness (Wu et al., 2023). This makes PILOT particularly well-suited for evaluating real-world intervention outcomes in diverse educational contexts.

Research on the PILOT (Positive Interpersonal and Life Orientation Training) program has demonstrated that participants with higher exposure levels, defined as attending at least 60% of the sessions, exhibited greater resilience, enhanced psychological flourishing, and improved self-efficacy in resisting substance use, along with a reduction in internet addiction tendencies (Wu et al., 2023). However, there remains a lack of empirical studies exploring PILOT’s influence on broader developmental outcomes, particularly in areas like inhibitory control. This shortfall limits our understanding of the program’s potential contributions to cognitive development in adolescence. Investigating this area is essential for gaining a more complete picture of PILOT’s impact. Expanding the evidence base to include cognitive and regulatory outcomes could offer valuable direction for enhancing school-based mental health strategies and shaping educational practices that support adolescents’ psychological and self-regulatory development.

### 1.3 Objective of this study

To evaluate the effectiveness of the PILOT program in enhancing executive functions, this study recruited first-year junior high school students (average age 13.37)—an age marked by ongoing neurodevelopment and maturation of inhibitory control (Cai et al., 2019). An intervention-based design was employed to examine the differences in inhibitory control between an intervention group and a control group.

The study had three aims. The first was to examine whether students receiving the program showed greater pre-to-post improvement in counting Stroop tasks than students in the control group, and whether such an improvement was specific to incongruent trials compared to congruent trials. The second was to examine whether the improvement in counting Stroop tasks was accompanied by measurable changes in psychological well-being over the same period to those who participated in the program. The third was to examine whether changes in Stroop interference were associated with concurrent changes in well-being within the program group.

## 2 Materials and methods

### 2.1 Participants

Participants were 7th-grade junior high school students from eight different schools in Keelung City and Yunlin County. Allocation was carried out at the school level rather than at the level of individual students, and was not random: schools were recruited by the local education department, and those willing to deliver the program formed the intervention group while the remaining schools served as controls. Four schools delivered the Positive Interpersonal and Life Orientation Training (PILOT) program (PILOT group, *n* = 404), and four continued with their usual study routines (control group, *n* = 274). Because whole schools were allocated, no school contributed students to both groups. The study is therefore a non-randomized controlled trial. The research project was approved by the National Taiwan Normal University Research Ethics Committee.

Due to the practical reasons, this study was carried out over two academic years, forming two independent implementation cohorts of different students. Cohort 1 (March 2023 to January 2024) comprised 303 PILOT students from three schools and 122 control students from two schools; Cohort 2 (March 2024 to January 2025) comprised 101 PILOT students from two schools and 152 control students from three schools. Two schools took part in both years, one in the PILOT group and one in the control group on both occasions, no school changed groups between cohorts and no student took part twice.

A total of 715 participants were initially recruited. Because the task was a four-alternative forced-choice task, chance performance was 0.25; accuracy below 0.20 (applied at both pre-test and post-test) was therefore taken to indicate that a participant was not following instructions. Of these, 24 were excluded for inattentiveness and 13 were excluded for excessive missing data (missing more than 50% of trials). These 37 exclusions did not differ in rate between groups (PILOT 26/430, 6.0%; control 11/285, 3.9%), χ^2^(1) = 1.67, *p* = 0.196. Consequently, the final sample comprised 678 participants: 404 in the PILOT group (185 male, mean age = 13.32) and 274 in the control group (124 male, mean age = 13.41). 2.2 procedures

Each cohort was followed for approximately 12 months, from the second semester of Grade 7 through the first semester of Grade 8. Students in the intervention group received the PILOT program, while students in the control group followed their standard school curriculum prescribed by Taiwan’s 12-Year Basic Education Curriculum Guidelines during the corresponding period and received no component of the PILOT program. Both groups completed a counting Stroop task before and after the intervention to assess inhibitory control.

### 2.2 Positive interpersonal life orientation training (PILOT) intervention

The PILOT curriculum comprises eight modules, delivered by class teachers to intact classes within regular classroom settings as part of the ordinary school timetable rather than as an additional or extracurricular activity. The modules are delivered as sixteen 45-min sessions, eight in each semester; scheduling within a semester was determined by the class teacher. Teachers received a six-hour workshop before implementation together with an additional summer training session, led by the research team and by experienced PILOT teachers, covering the aims of the program and a rehearsal of the lesson plan for each unit. Eight core learning units are described below.

#### 2.2.1 Social skills

Students learn the elements of effective communication—clarity and consistency—and techniques to avoid misunderstandings, such as restating and questioning. They practice sending and receiving messages during group activities, note down facts, feelings, findings, and future actions to improve misunderstanding situations, and engage in role-playing to consider reactions to misunderstandings.

#### 2.2.2 Communication skills

Students learn to interact with different people using both verbal and nonverbal communication skills. They play games involving asking questions and learn about different factors that lead to effective communication.

#### 2.2.3 Anger management

This educational approach teaches students to manage their anger by breaking down their experiences into four analytical components: Fact, Feeling, Finding, and Future. Through practicing real-life situations, they learn how different ways of expressing anger—such as acting properly (assertively), having an outburst (aggressively), or suppressing it (internally)—can either benefit or harm their well-being across physical, psychological, and social aspects of life.

#### 2.2.4 Anxiety management

Students learn to be aware of and manage their anxiety when facing new environments and challenges. Techniques include deep breathing, relaxation, reevaluating thoughts, practicing in advance, and seeking help. They are encouraged to note down their anxiety situations and their feelings after learning management skills in the PILOT course.

#### 2.2.5 Decision making

Students learn to recognize choices in life, analyze factors influencing decision-making, and use the “Clarify-Consider-Choose” method to make better judgments and decisions. They also self-rate their decision-making styles via a questionnaire.

#### 2.2.6 Assertiveness

Students practice communication skills for dealing with differing opinions to avoid coercion or belittling others. They learn about their communication styles in annoying situations and role-play scenarios to practice assertively rejecting inappropriate requests.

#### 2.2.7 Conflict resolution

Students learn six steps (stay calm, calm the other person, listen to what the other person says, stand firm in your position, show respect, solve the problem) for resolving conflicts and practice these steps in role-playing situations.

#### 2.2.8 Resisting peer pressure

Students learn eight different rejection skills during this session. They are encouraged to note down their reactions based on these skills when facing peer pressure.

### 2.3 Questionnaires

The Psychological Well-Being Scale (PWBS) is an 84-item self-report inventory developed by Ryff to assess six dimensions of psychological well-being. In this study, we adopted a shortened Chinese version consisting of 18 items, with three items for each of the six dimensions: (1) Positive Relations with Others (e.g., I enjoy personal and mutual conversations with family members or friends); (2) Autonomy (e.g., I have confidence in my opinions, even if they are contrary to the general consensus); (3) Environmental Mastery (e.g., In general, I feel I am in charge of the situation in which I live); (4) Personal Growth (e.g., I think it is important to have new experiences that challenge how I think about myself and the world); (5) Purpose in Life (e.g., I enjoy making plans for the future and working to make them a reality); (6) Self-Acceptance (e.g., When I look at the story of my life, I am pleased with how things have turned out). The short-form Chinese version of the questionnaire has demonstrated good internal consistency, reliability, and content validity (Li, 2014). Participants rated each item on a 6-point Likert scale ranging from 1 (never or very rarely true) to 6 (almost always or always true). Dimension scores were calculated by summing the three items for each construct, so that each subscale ranges from 3 to 18, with higher scores indicating greater psychological well-being.

### 2.4 Behavioral task

In our current study, the counting Stroop task was employed to evaluate inhibitory control in both two groups of participants. In the counting Stroop task (Fan et al., 2017), there were three experimental stimuli: congruent, incongruent, and control conditions (see Figure 1). In the ‘congruent’ condition, the number of Chinese characters aligned with the meaning those characters represented, including ‘one,’ ‘two,’ ‘three,’ or ‘four.’ (i.e., ‘一’, ‘二’, ‘三’, ‘四’ in Chinese). In the ‘incongruent’ condition, the number of Chinese characters did not correspond with the meaning of the characters. In the ‘control’ condition, the selected Chinese characters conveyed meanings unrelated to numbers (i.e., ‘不’ meaning ‘no’; ‘人’ meaning ‘people’). For all the stimuli selected, we matched each word based on specific characteristics, including the number of syllables, visual complexity (strokes), and usage. Participants were required to respond to the number of stimuli by pressing the corresponding key on the keypad, utilizing the four buttons (1, 2, 3, 4 buttons arranged from left to right). They were instructed to respond as quickly as possible without compromising accuracy, using their index and middle fingers for the responses.

**Figure 1:**
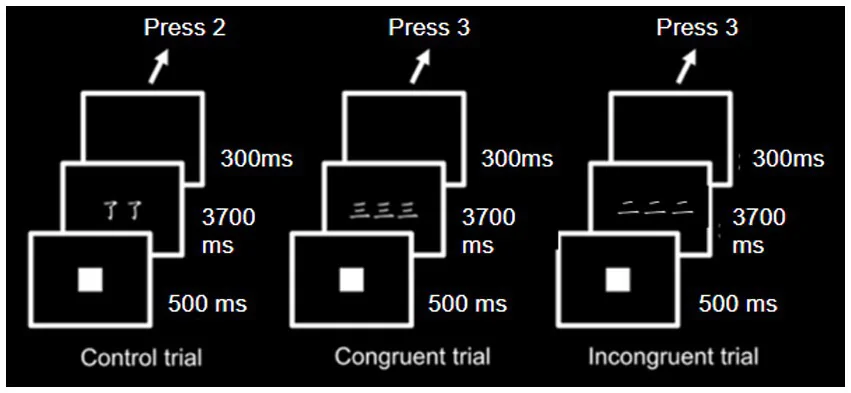
The three conditions on the counting Stroop task. Conditions differed depending on whether the number of words aligned with (congruent conditions), conflicted with (incongruent conditions), or had no relationship (control conditions) with the meaning of the word.

### 2.5 Data analysis

The counting Stroop Task: A three-way ANOVA was employed with 2 groups (PILOT, control) by 2 times (pre, post) by 3 conditions (congruent, incongruent, control). For the counting Stroop task, the data included the percentage of correct responses (accuracy) and the speed of response (reaction time). For reaction time analysis, incorrect trials were excluded, focusing solely on the correct trials. Additionally, outliers were eliminated, defined as values at least 2.5 standard deviations from the mean for each condition and each participant (Lee et al., 2023). Because students were tested twice and were nested within the eight participating schools, the analysis was also refitted as a linear mixed model with random intercepts for school and for participants.

The Psychological Well-Being Scale: Change scores (post minus pre) were computed for each subscale and compared between groups. Within the PILOT group, linear regression was used to examine the association between change in Stroop interference and change in each subscale over the same interval. Because six subscales were tested, *p*-values were corrected using the Holm-Bonferroni procedure.

## 3 Results

### 3.1 Participants

There were no significant age differences between the PILOT group and the control group (Table 1). The two groups also did not differ at baseline on adverse childhood experiences, resilience, parent–child relationship, peer relationship, school adjustment, or family structure (all *ps* > 0.27), nor did baseline psychological well-being differ (PILOT M = 76.82, control M = 77.43, *p* = 0.63).

**Table 1:** Demographic information on two groups of participants.

Gender, *n*	PILOT	Control	*p*
Male	185	124	> 0.05
Female	219	150	
Mean age, Mean (SD)	13.32 (0.96)	13.41 (0.50)	> 0.05
Age range (years old)	12 ~ 14	12 ~ 14	

PILOT, participants in the intervention group; control, participants in the control group. Data are given as mean (standard deviation).

### 3.2 The counting Stroop task

The results for each participant, including accuracy and reaction time, were collected during the counting Stroop task at two time points: before and after the PILOT intervention (see Table 2). For accuracy, there was a significant main effect for condition (*F*(2, 1,352) = 270.61, *p* < 0.001, *η^2^* = 0.026) and time (*F*(1, 676) = 29.56, *p* < 0.001, *η^2^* = 0.014). Two significant interactions were found: time × group (*F*(1, 676) = 4.49, *p* < 0.05, *η^2^* = 0.002) and time × condition (*F*(2, 1,352) = 14.62, *p* < 0.001, *η^2^* = 0.001). Further analyses of the time × group interaction revealed that the PILOT group showed higher accuracy on post-training compared to pre-training (*F*(1, 676) = 33.42, *p* < 0.001), while the control group also showed significantly higher accuracy on post-training compared to pre-training (*F*(1, 676) = 5.04, *p* < 0.05) (see Figure 2). Further analysis of the time × condition interaction indicated that participants responded with greater accuracy in incongruent trials on post-training compared to pre-training (*F*(1, 1,352) = 43.17, *p* < 0.001), as well as in congruent trials (*F*(1, 1,352) = 14.89, *p* < 0.001) and control trials (*F*(1, 1,352) = 18.45, *p* < 0.001). Additionally, the Time × Condition × Group interaction was not significant, *F*(2, 1,352) = 0.79, *p* = 0.453, nor were the condition × group interaction, *F*(2, 1,352) = 1.02, *p* = 0.362, or the main effect of group, *F*(1, 676) = 0.01, *p* = 0.92.

**Table 2:** Counting Stroop task performance of accuracy and reaction time (in millisecond).

Group	Time	Congruent		Incongruent		Control	
		Mean	SD	Mean	SD	Mean	SD
Accuracy							
PILOT (*n* = 404)	pre	0.94	0.15	0.90	0.16	0.94	0.14
	post	0.98	0.07	0.94	0.09	0.97	0.07
Control (*n* = 274)	pre	0.96	0.11	0.90	0.14	0.95	0.12
	post	0.95	0.09	0.93	0.11	0.96	0.09
Reaction time							
PILOT (*n* = 404)	pre	1020.87	226.92	1094.31	229.32	1018.57	215.95
	post	916.45	193.93	985.07	201.12	911.12	186.79
Control (*n* = 274)	pre	998.42	218.67	1076.92	280.37	985.33	219.00
	post	878.40	207.45	928.75	218.80	871.50	215.59

PILOT, participants in the intervention group; control, participants in the control group.

**Figure 2:**
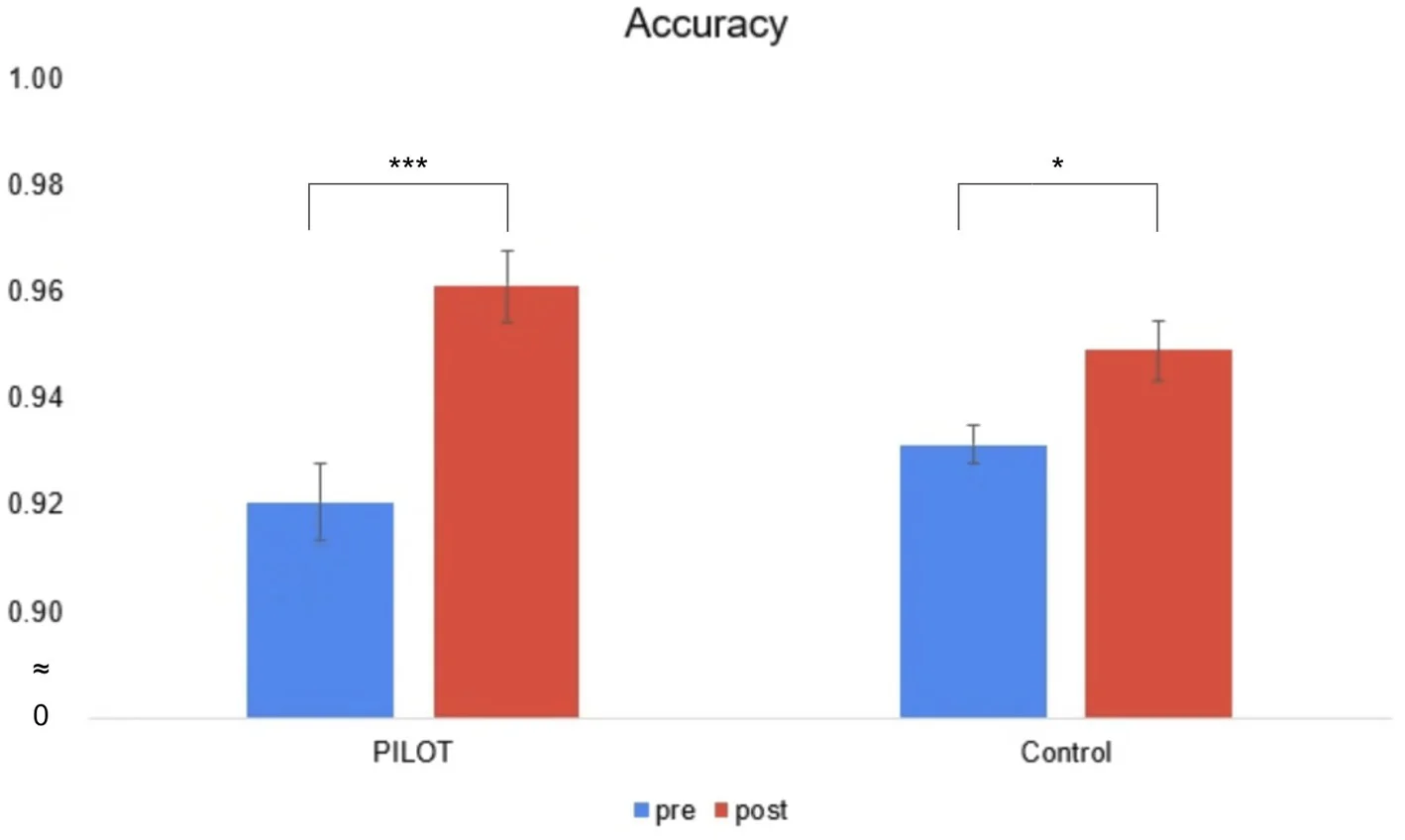
The mean difference in accuracy between pre- and post-PILOT training was examined for participants in the intervention group (*n* = 404) and the control group (*n* = 274). Results revealed a significant improvement in accuracy in the intervention group compared to the control group, indicating that the PILOT intervention effectively enhanced performance during the counting Stroop task.

Because students were tested twice and were nested within schools, accuracy was also analyzed as a linear mixed model with random intercepts for school and for participants. The Time × Condition × Group interaction remained non-significant, *F*(2, 3,380) = 0.28, *p* = 0.75, but the Time × Group interaction remained significant, *F*(1, 3,380) = 16.11, *p* < 0.001.

Regarding reaction time, a main effect for time (*F*(1, 676) = 175.81, *p* < 0.001, *η^2^* = 0.064), a main effect for condition (*F*(2, 1,352) = 217.11, *p* < 0.001, *η^2^* = 0.021) and a main effect for group (*F*(1, 676) = 6.93, *p* < 0.01, *η^2^* = 0.005) were significant. A significant time × condition interaction was found, (*F*(2, 1,352) = 3.13, *p* < 0.05, *η^2^* < 0.01). Simple main effect analyses revealed that participants responded with significantly faster reaction times in the post-training phase compared to pre-training across all conditions: incongruent trials (*F*(1, 1,352) = 141.66, *p* < 0.001), congruent trials (*F*(1, 1,352) = 132.66, *p* < 0.001), and control trials (*F*(1, 1,352) = 142.67, *p* < 0.001). Other than the significant results mentioned, there were no additional significant interactions (*ps* > 0.05).

### 3.3 Associations between change in Stroop interference and change in well-being

Linear regression analyses were conducted to examine whether Stroop interference at Time 2 minus Time 1 (calculated as accuracy in incongruent trials minus congruent trials) was associated with the improvements in psychological well-being (PWBS) within the PILOT group. The dependent variables were the difference scores on each PWBS subscale (Time 2 minus Time 1), reflecting changes in well-being following the intervention. Because six subscales were tested, *p*-values were corrected using the Holm-Bonferroni procedure. Significant regression models were found for three subscales: Autonomy: *F*(1, 373) = 6.86, *p* < 0.01, corrected *p* = 0.037, Adjusted *R^2^* = 0.015; Environmental Mastery: *F*(1, 373) = 13.19, *p* < 0.001, corrected *p* = 0.002, Adjusted *R^2^* = 0.032 and Self Acceptance: *F*(1, 373) = 8.86, *p* < 0.01, corrected *p* = 0.016, Adjusted *R^2^* = 0.021 (see Figures 3–5 and Table 3).

**Figure 3:**
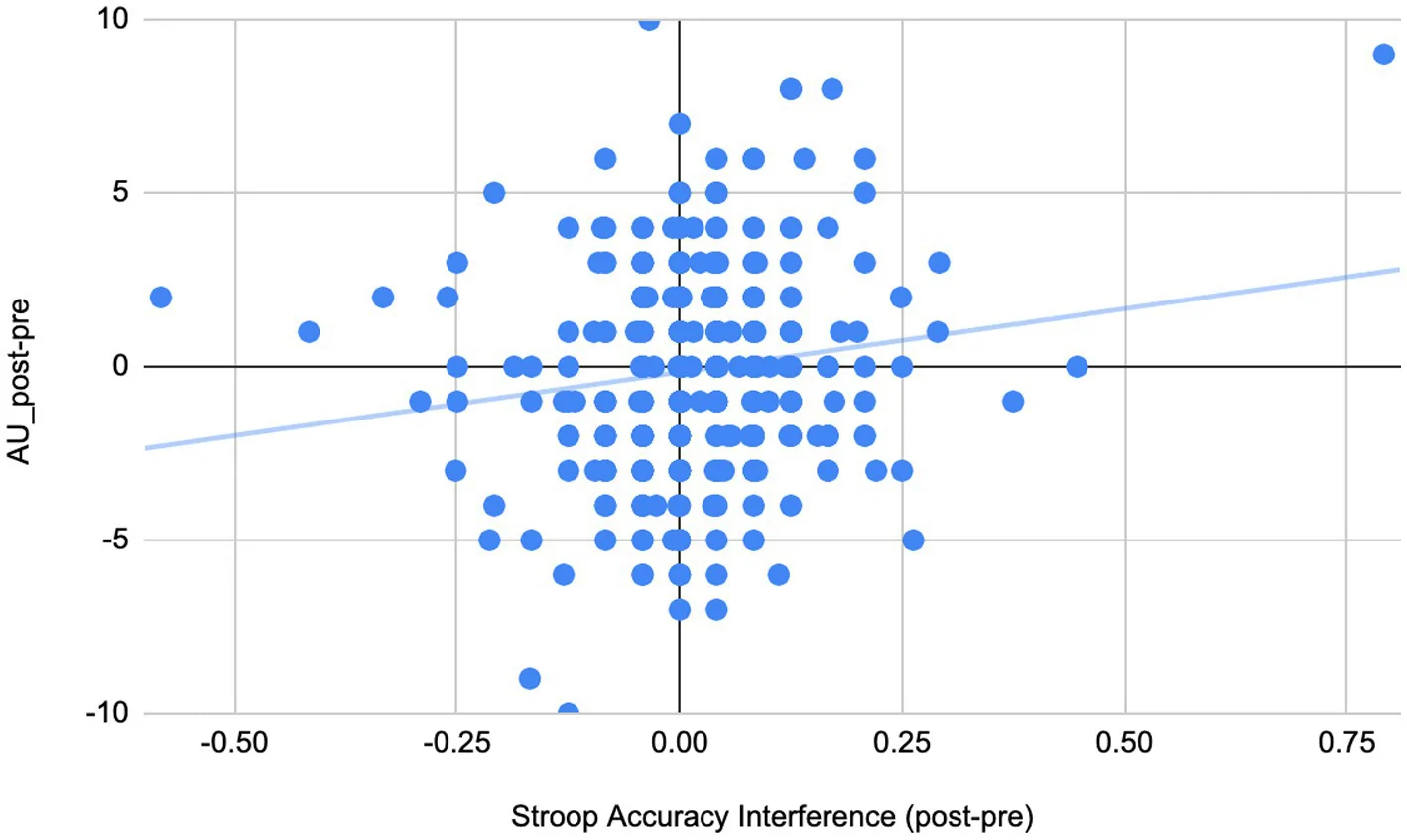
Relationship between (post-pre) Stroop interference and changes in Autonomy scores for the PILOT group. The regression analysis showed that increases in Stroop interference (post-pre) was significantly associated with greater improvements in Autonomy.

**Figure 4:**
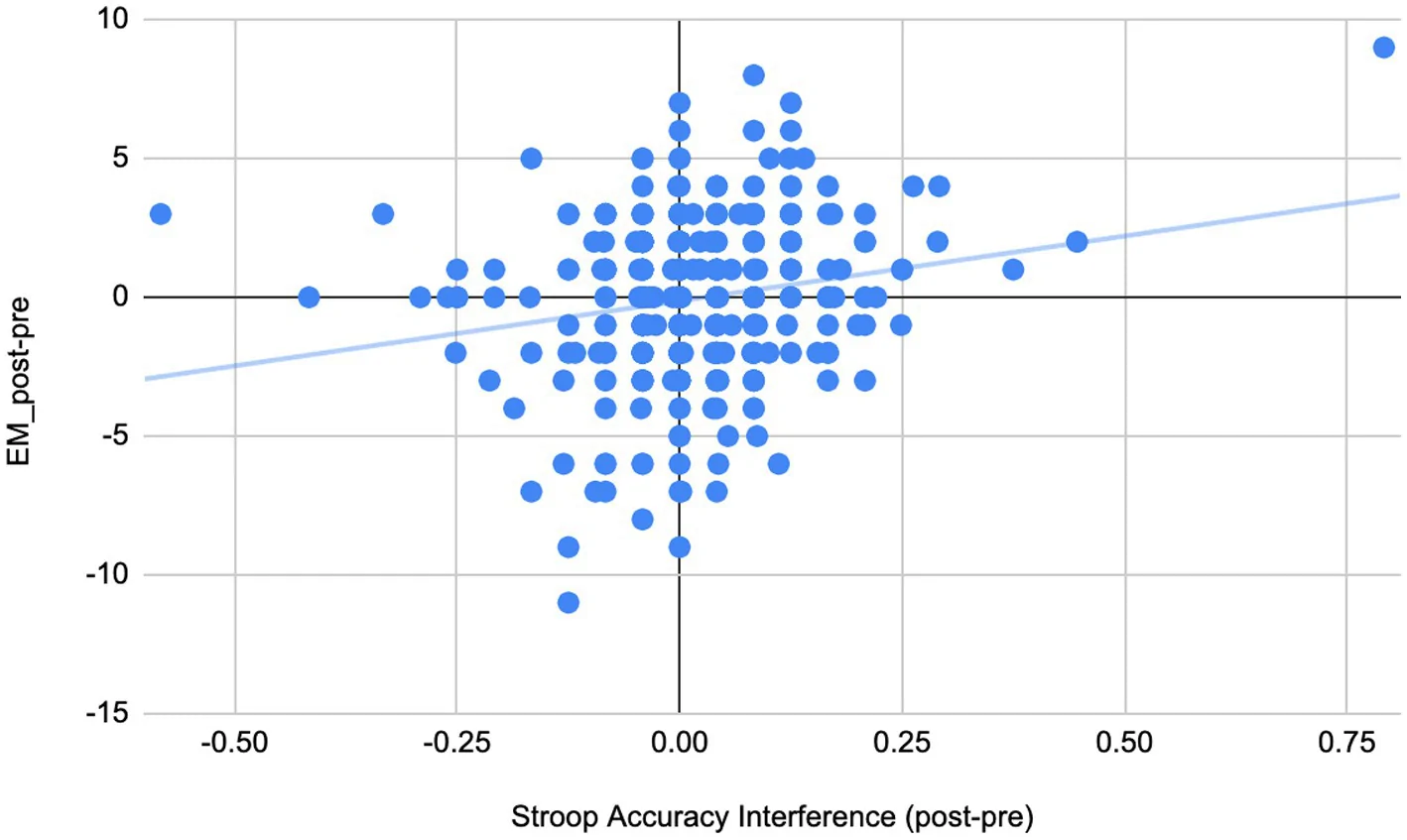
Relationship between (post-pre) Stroop interference and changes in Environmental Mastery scores for the PILOT group. The regression analysis showed that increases in Stroop interference (post-pre) was significantly associated with greater improvements in Environmental Mastery.

**Figure 5:**
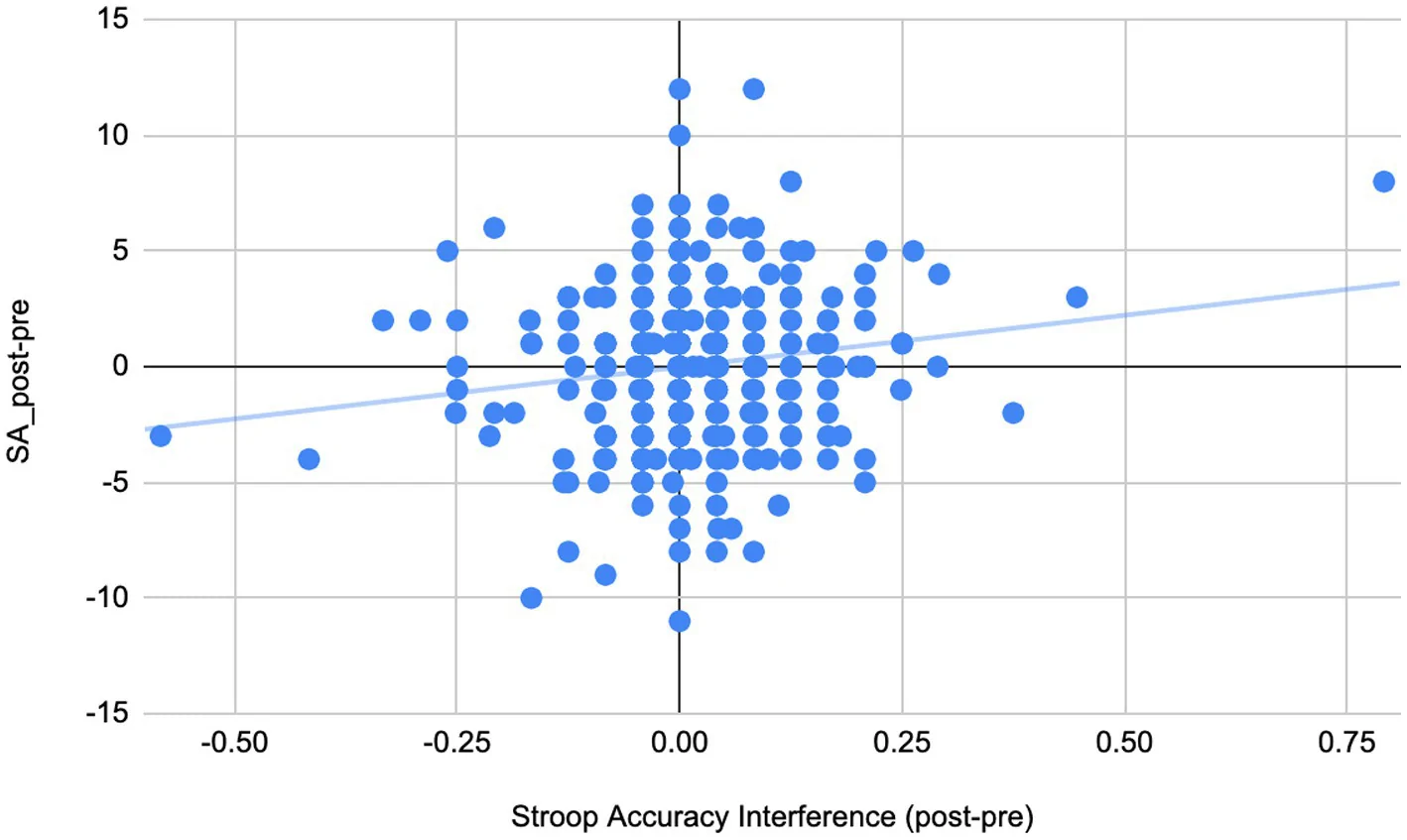
Relationship between (post-pre) Stroop interference and changes in self acceptance scores for the PILOT group. The regression analysis showed that increases in Stroop interference (post-pre) was significantly associated with greater improvements in self acceptance.

**Table 3:** Associations between change in Stroop interference and change in psychological well-being within the PILOT group.

Subscale	*B* (*SE*)	β	95% CI	*F*(1, 372)	*p*	Holm *p*	Adj. *R*^2^
Positive Relations with Others	1.76 (1.27)	0.071	[−0.75, 4.26]	1.90	0.169	0.236	0.002
Autonomy	3.64 (1.39)	0.135	[0.91, 6.37]	6.86	0.009*	0.037*	0.015
Environmental Mastery	4.71 (1.30)	0.185	[2.16, 7.26]	13.19	< 0.001*	0.002*	0.032
Personal Growth	2.10 (1.34)	081	[−0.54, 4.74]	2.46	0.118	0.236	0.004
Purpose in Life	2.48 (1.40)	0.091	[−0.27, 5.23]	3.14	0.077	0.232	0.006
Self-Acceptance	4.44 (1.49)	0.153	[1.51, 7.37]	8.86	0.003*	0.016*	0.021

The predictor is change in Stroop interference (post minus pre, accuracy on incongruent minus congruent trials); outcomes are change scores (post minus pre) on each psychological well-being subscale over the same interval. Holm p = p-value corrected across the six models using the Holm–Bonferroni procedure.

### 3.4 Questionnaires: the Psychological Well-Being Scale (PWBS)

Subscale scores of the Psychological Well-Being Scale were calculated to examine participants’ well-being across different dimensions (see Table 4). To test directly whether the intervention affected psychological well-being, change scores on each PWBS subscale were compared between groups. Because Environmental Mastery, Self-Acceptance, and Autonomy were the three subscales related to the changes in Stroop performance, *p*-values were corrected across these three using the Holm-Bonferroni procedure. Self-Acceptance showed a significant Time × Group interaction, *F*(1, 625) = 6.78, *p* = 0.009, corrected *p* = 0.028, *d* = 0.21. Simple main effects of time showed that self-acceptance declined in the control group, *F*(1, 252) = 5.75, *p* = 0.017, but did not change in the PILOT group, *F*(1, 373) = 0.87, *p* = 0.352. Simple main effects of group did not differ in the pre-test, *F*(1, 625) = 0.46, *p* = 0.494, but the two groups reached significance in the post-test, *F*(1, 625) = 3.62, *p* = 0.05. Environmental Mastery and Autonomy did not show a Time × Group interaction. Full results are given in Table 5.

**Table 4:** PWBS scores on two groups and two time points.

PWBS	PILOT pre	PILOT post	Control pre	Control post
Positive relations with others	13.10 (0.14)	12.92 (0.14)	13.24 (0.18)	13.04 (0.19)
Autonomy	12.45 (0.14)	12.45 (0.14)	12.40 (0.22)	12.42 (0.21)
Environmental mastery	13.21 (0.14)	13.21 (0.15)	13.45 (0.20)	13.51 (0.19)
Personal growth	13.51 (0.14)	13.60 (0.15)	13.63 (0.21)	13.64 (0.20)
Purpose in life	12.69 (0.17)	12.76 (0.16)	12.65 (0.23)	12.69 (0.21)
Self-acceptance	11.82 (0.17)	11.98 (0.17)	12.20 (0.23)	11.46 (0.23)

PILOT, Positive Interpersonal Life Orientation Training; PWBS, Psychological Well-Being Scale. Data are given as mean (standard error).

**Table 5:** Between-group comparison of change in psychological well-being.

Subscale	PILOT M (SD)	Control M (SD)	F	df	*p*	Holm p	d
Positive Relations with others	−0.18 (2.88)	−0.21 (2.90)	0.01	1, 625	0.920	-	+0.01
Autonomy	+0.00 (3.11)	+0.02 (3.72)	0.01	1, 625	0.943	1.00	−0.01
Environmental mastery	−0.00 (2.87)	+0.04 (3.15)	0.03	1, 623	0.861	1.00	−0.01
Personal growth	+0.07 (2.94)	+0.01 (3.01)	0.07	1, 624	0.795	-	+0.02
Purpose in life	+0.09 (3.07)	+0.02 (3.41)	0.07	1, 625	0.794	-	+0.02
Self-acceptance	+0.15 (3.11)	−0.57 (3.78)	6.78	1, 625	0.009	0.028	+0.21

Values are change scores (post minus pre). PILOT *n* = 374, control *n* = 253. Residual degrees of freedom vary slightly because two participants missed one answer on a single subscale. Holm p = p-value corrected across Environmental Mastery, Self-Acceptance, and Autonomy, the three subscales identified in Table 3; the remaining subscales are reported without correction.

## 4 Discussion

To our knowledge, this study is the first to examine the longitudinal effects of the Positive Interpersonal Life Orientation Training (PILOT) program on both inhibitory control and psychological well-being among seventh-grade adolescents. Students in the PILOT group showed greater pre-to-post improvement in overall counting Stroop accuracy than students in the control group; the improvement was not specific to incongruent trials. Within the PILOT group, change in Stroop interference was associated with concurrent change in three dimensions of psychological well-being.

These findings suggest that life skills training embedded within school curricula may serve as an effective tool for enhancing both cognitive functioning and associated with broader psychological well-being, further highlighting the interdependence between cognitive regulation and emotional development during early adolescence.

### 4.1 The results of the PILOT intervention

In this study, the PILOT intervention emphasized eight core components of life skills training: Social Skills, Communication Skills, Anger Management, Anxiety Management, Decision Making, Assertiveness, Conflict Resolution, and Resisting Peer Pressure. At first glance, these elements may appear to focus primarily on emotional regulation and interpersonal competence. However, a closer examination reveals that many of these life skills inherently draw upon and strengthen the core processes of cognitive ability and self-regulation. For instance, adolescents are particularly susceptible to peer pressure and emotional reactivity, such as sudden outbursts of anger (Pfeifer et al., 2011). Through training in anger management, students are encouraged to pause before reacting, recognize their emotional triggers, and select more adaptive responses, skills that require them to control automatic or impulsive behaviors. Similarly, in the decision-making module, students are introduced to the framework of “Clarify–Consider–Choose,” which promotes deliberate thinking and discourages impulsive, intuition-based responses. In order to apply this model effectively, students must resist the urge to act on their initial impulses and instead engage in reflective processing, an essential aspect of utilizing their cognitive ability. Thus, although the life skills taught in the PILOT program are framed in several emotional and social terms, they could foster the development of cognitive ability following the intervention.

The notion that training programs not explicitly designed to enhance inhibitory control can nonetheless yield improvements in this domain has been supported by several studies. For example, Karbach and Kray (2009) demonstrated that task-switching training led to measurable gains in inhibition. Kattner (2021) further showed that transfer effects depend on the extent to which inhibitory demands are embedded in the training task. Their findings revealed that a modified dual n-back task requiring participants to suppress automatic responses reduced interference from irrelevant auditory stimuli, whereas the standard dual n-back task, lacking such inhibitory demands, did not. These results suggest that improvements in inhibition may emerge when cognitive training engages inhibitory processes, even if inhibition is not the primary target. However, other studies have reported inconsistent transfer effects across cognitive domains, most likely because task-specific paradigms tend to promote narrow learning without generalization (Allom et al., 2016; Takacs and Kassai, 2019). By contrast, the present study employed the PILOT intervention, which is situated within a broader framework of cognitive skills rather than a single trained task. Here the PILOT group improved more than the control group in overall counting Stroop accuracy, even though the advantage was of comparable magnitude across congruent, control, and incongruent trials so the interference effect did not differ between groups. Training embedded in everyday situations may therefore affect task performance more broadly, without producing the interference-specific transfer that Kattner (2021) obtained when inhibitory demands were built directly into the trained task.

Notably, this study also found significant improvements in counting Stroop accuracy within the control group, suggesting that cognitive ability naturally develops over time during early adolescence, likely due to ongoing brain maturation and cognitive growth. This finding aligns with existing developmental research indicating that executive functions, including inhibition, continue to improve throughout adolescence as the prefrontal cortex matures (Jaeger, 2013). However, the fact that the intervention group demonstrated even greater gains beyond those observed in the control group suggests that the PILOT program contributed additional benefits beyond what could be attributed to biological development alone.

### 4.2 The relationship between the improvement of inhibitory control and psychological well-being

Our findings suggest improvements in inhibitory control associated with subsequent gains in psychological well-being. Adolescents who showed greater enhancement in inhibitory control during the PILOT training demonstrated larger increases in autonomy, environmental mastery and self acceptance. These results align with prior research linking cognitive control to well-being. For instance, Lee and Chao (2012) found that stronger active inhibition—specifically, the ability to suppress emotionally distracting information—was associated with higher levels of mindfulness, which in turn supported psychological well-being. Mindfulness, defined as a present-centered and non-judgmental awareness of one’s internal experiences (Bishop et al., 2004), is widely regarded as a mechanism for improving attention and regulating emotions (Cásedas et al., 2020). Individuals with stronger inhibitory control may be better equipped to maintain mindfulness by filtering out irrelevant stimuli and moderating unwanted emotional reactions, thereby promoting emotional stability, life satisfaction, and reduced vulnerability to depressive symptoms (Lee and Chao, 2012).

Other research has also highlighted the importance of inhibitory control for mental health. One study examining young adults under stress used both non-emotional (scenic pictures) and emotional (facial expressions) Go/No-Go tasks to assess inhibitory control. Their results indicated that higher resilience was associated with stronger non-emotional inhibitory control, whereas higher psychological distress and anxiety were linked to poorer emotional inhibitory control (Afek et al., 2021). Although additional work is needed to clarify why emotional and non-emotional inhibitory control relate differently to aspects of well-being, the existing evidence collectively underscores the central role of cognitive control in associating with emotion regulation and, ultimately, psychological health.

In our study, we found that the primary three dimensions of psychological well-being—autonomy, environmental mastery and self acceptance—were associated with the extent of improvements in inhibitory control. One possible explanation is that these three aspects are conceptually and empirically interrelated. Prior research has shown that dimensions of psychological well-being, as measured by Ryff’s Psychological Well-Being Scale, are closely tied to facets of autonomy assessed through the EDATVA scale (cited by De-Juanas et al., 2020). Significant correlations have been observed between self-organization and environmental mastery, suggesting that the ability to structure one’s goals and effectively manage the environment is strongly linked to higher psychological functioning (cited by De-Juanas et al., 2020). These findings indicate that inhibitory control may serve as a foundational self-regulatory capacity that underpins autonomy, environmental mastery and self acceptance. Future research should investigate whether improvements in inhibitory control exert domain-specific effects on psychological well-being and explore the potential mediating role of self-regulation and emotional processes in these associations.

### 4.3 Group differences in psychological well-being

The two groups differed in the self-acceptance domain. Over the twelve-month interval, self-acceptance declined significantly in the control group but not in the PILOT group. This change pattern in the control group is in line with the development of well-being, such as self-esteem, in early adolescence (Katsantonis et al., 2023). Early adolescence brings simultaneous biological, social, and educational changes, including the transition to secondary schooling, heightened sensitivity to peers, and rising academic demands. Katsantonis et al. (2023) followed early adolescents (from age 11 to 14) in the UK and found that subjective well-being declined substantially over this interval. The decline was concentrated in satisfaction with school, schoolwork, and friendships, which the authors would attribute to the greater demands of secondary education and to changes in peer relationships. The same downward trend appears cross-nationally. Comparing cohorts of 15-year-olds, Marquez and Long (2021) reported that mean life satisfaction fell in 39 of 46 countries between 2015 and 2018, across a range of sociodemographic groups.

Of the six domains, self-acceptance may be the one most exposed at this age, because it is the one that turns on how adolescents evaluate themselves—and in early adolescence that evaluation is made increasingly by reference to peers, whose opinions and relative standing carry more weight than at earlier ages. Ruan et al. (2023) found that this comparison carries a cost: social comparison mediated the association between adolescents’ self-acceptance and depressive symptoms. Although the pilot curriculum in this study has no unit directly devoted to self-acceptance, several of its modules do, however, ask students to recognize their own reactions and act on them rather than suppress them. Students in Resisting Peer Pressure rehearse refusing a group when refusing is uncomfortable, and practice staying with that discomfort instead of giving way; Anger Management and Anxiety Management make the same demand in other situations. Repeated practice of this kind may help students hold a steady view of themselves through a period in which it typically declines.

## 5 Limitations

First, an important limitation of the present study is that it does not address the long-term sustainability of the observed improvements. Although the findings are consistent with previous intervention studies demonstrating short-term gains on tasks such as the Stop-Signal Task, Go/No-Go, and Stroop, particularly among adolescents (Karbach and Unger, 2014), the extent to which these gains endure over time remains unclear. Berkman et al. (2014) further argue that these improvements may stem from temporary shifts in neural activation rather than lasting structural changes. In particular, increased activation and functional connectivity in areas such as the right inferior frontal gyrus and dorsolateral prefrontal cortex, key regions for executive control, may support immediate task performance without producing durable, generalized benefits. Future research should explore whether interventions like PILOT produce lasting neurocognitive changes and broader behavioral outcomes, especially when delivered in more intensive or longitudinal formats.

A promising avenue for future research is to more explicitly incorporate the emotional dimensions of the PILOT training. While the present study primarily emphasized students’ inhibitory control, the intervention also included elements targeting emotional regulation and interpersonal skills. Extending outcome measures to include emotional inhibitory-control paradigms would allow researchers to better examine how training shapes the dynamic interaction between affective processes and cognitive functioning. Prior work has underscored the close interplay between emotional and cognitive abilities, yet the directionality of this relationship remains debated, whether improvements in emotional regulation facilitate gains in inhibitory control, or vice versa (Bartholomew et al., 2021). By systematically integrating both cognitive and emotional indices, future studies could provide a more comprehensive understanding of how PILOT fosters self-regulation, thereby clarifying whether enhancements in inhibitory control contribute to greater emotional resilience, more adaptive decision-making, and stronger social adjustment.

## Data Availability

The original contributions presented in the study are included in the article/supplementary material, further inquiries can be directed to the corresponding author.
